# Optically Active TiO_2_:Er Thin Films Deposited by Magnetron Sputtering

**DOI:** 10.3390/ma14154085

**Published:** 2021-07-22

**Authors:** Anna Kot, Marta Radecka, Dominik Dorosz, Katarzyna Zakrzewska

**Affiliations:** 1 Faculty of Materials Science and Ceramics, AGH University of Science and Technology, al. A. Mickiewicza 30, 30-059 Kraków, Poland; radecka@agh.edu.pl (M.R.); ddorosz@agh.edu.pl (D.D.); 2 Faculty of Computer Science, Electronics and Telecommunications, AGH University of Science and Technology, al. A. Mickiewicza 30, 30-059 Kraków, Poland; zak@agh.edu.pl

**Keywords:** photoluminescence, up-conversion, titanium dioxide, erbium ions, thin films, hydrogen generation by water splitting, photoanode materials

## Abstract

Titanium dioxide photoanodes for hydrogen generation suffer from a profound mismatch between the optical absorption of TiO_2_ and the solar spectrum. To solve the problem of low solar-to-chemical efficiency, optically active materials are proposed. In this work, TiO_2_ thin films containing erbium were deposited by radio frequency RF magnetron sputtering under ultrahigh vacuum conditions UHV. Morphology, structural, optical and electronic properties were studied. TiO_2_:Er thin films are homogenous, with uniform distribution of Er ions and high transparency over the visible VIS range of the light spectrum. However, a profound 0.4 eV blue shift of the fundamental absorption edge with respect to undoped TiO_2_ was observed, which can be attributed either to the size effect due to amorphization of TiO_2_ host or to the onset of precipitation of Er_2_Ti_2_O_7_ nanocrystals. Near-infrared NIR to VIS up-conversion is demonstrated upon excitation at 980 nm, while strong green photoluminescence at 525 and 550 nm occurs upon photon absorption at 488 nm.

## 1. Introduction

Optically active materials that exhibit either down or up-conversion of light frequency are gaining rapidly new and versified fields of applications in optoelectronics, photonics, bioengineering and photocatalysis [1,2,3,4,5,6,7]. Luminescence from glasses containing lanthanide ions is widely exploited in sensor devices, solid-state lasers, telecommunication fibers, displays, plasma monitors, solar cells, optical thermometers, etc. [1,2,3,8,9].

Down- and up-conversion belong to a group of nonlinear optical processes and can be classified as light management techniques [10]. Down- and up-converting materials are usually based on rare earth RE ions incorporated in host matrices, i.e., phosphors [1]. Here, the choice of particular RE ions remains a key factor, but the properties of the host material are equally important for the efficiency of the process.

Contrary to the well-known optically active glasses [11], titanium dioxide is not so frequently encountered as a host because of much higher phonon energy (<700 cm^−1^) contributing to non-radiative relaxation [12,13]. Nevertheless, TiO_2_ being a non-toxic wide-band-gap semiconductor with a relatively high refractive index and excellent stability in harsh environments remains one of the most extensively studied metal oxides with a vast variety of applications in photocatalysis and photoelectrochemistry [14,15]. However, low solar-to-chemical conversion efficiency due to the profound mismatch between the optical absorption of TiO_2_ and the solar spectrum has incited a lot of efforts towards improved light harvesting. Since the first demonstration of hydrogen generation from water splitting on TiO_2_ single crystal photoanodes in a photoelectrochemical cell PEC constructed by Fujishima and Honda [16], different doping schemes have been proposed. Substitutional doping for Ti^4+^ with metals Me in cation sublattice, O^−2^ ions with nitrogen N, carbon C or sulfur in anion sublattice, co-doping of both sublattices and non-stoichiometry of oxygen sublattice has led to at least four generations of photocatalysts and photoanode materials [17]. A serious drawback of these types of lattice modification manifests itself in an increased electron and hole recombination rate due to a build-up of additional states in the forbidden band gap of TiO_2_. An innovative approach based on the application of optically active materials aims at replacement of band gap engineering by “manipulation of light”.

The term “light manipulation” was invented for the purposes of efficient harvesting of the incident radiation in the case of solar cells [18]. It consists of either (1) photoluminesce when upon excitation with a photon of higher frequency, a lower frequency photon is emitted following the non-radiative transfer of an electron to a lower state; (2) down conversion involving two photons of higher energy to produce one photon of lower energy; or (3) up-conversion in which at least two low energy photons are absorbed in order to emit one photon of higher energy [18,19].

Titanium dioxide doped with lanthanide Ln-ions exhibits well-pronounced photoluminescence in the visible range of the light spectrum [20,21,22,23,24]. Numerous reports on the application of such nanomaterials in photoelectrochemical cells and photocatalytic decomposition of organic pollutants were published recently [25,26,27]. However, special attention should be paid to TiO_2_:Er in a thin film form because the planar structure is more suitable for photoanodes in PEC [28]. Morphology, crystal structure, phase composition and optical properties of thin films are profoundly affected by a chosen method of deposition. Photoluminescence measurements were carried out mainly for thin films grown by chemical methods [21,22,29]. Physical vapor deposition PVD techniques, including ion sputtering, are more reliable and yield more reproducible results. Therefore, in this work, we undertook the studies of undoped TiO_2_ and TiO_2_:Er thin films deposited by reactive radio frequency RF magnetron sputtering in ultra-high vacuum UHV system in order to assess the control of growth with appropriate composition and structure providing required optical properties.

Our previous research demonstrated improved performance of Ti(Nd)O_2_ photoanodes in combination with external up-converting glass [30]. Excitation with near-infrared (NIR) radiation (λ_exc_ = 940–980 nm), which results in the emission in the ultraviolet (UV) range (λ_em_ = 340 nm), has led to a 13-fold increase in the photocurrent [30].

Despite the extended research, there is no agreement concerning the mechanism of Er incorporation into TiO_2_ lattice and its effect on the optical properties of thin films. Even in the case of bulk glass-crystalline materials where the emission is conditioned by energy transfer mechanisms, including phonon-assisted phenomenon, up-conversion is strongly dependent on the RE ions localization [31]. It is generally accepted that the emission is highly sensitive to the local atomic and electronic structure of up-converting phosphors [27,32]. In order to become optically active, Er^3+^ should place itself in particular oxygen coordination and form oxygen bridges.

In this work, TiO_2_ thin films with incorporated Er were studied with the aim to correlate their morphological, structural and luminescent properties. The ultimate motivation of this research is to demonstrate that the design and preparation of a proper structure of TiO_2_ with embedded Er^3+^ ions will enable the up-conversion. It is believed that this procedure will improve the efficiency of solar-to-hydrogen conversion required for applications of TiO_2_:Er as photoanodes in PEC.

## 2. Materials and Methods

TiO_2_ thin films were deposited in the ultra-high vacuum UHV plant (PREVAC, Rogów, Poland) by the RF reactive magnetron sputtering from 2" Ti (purity 99.995%) and composite Ti/Er 90/10 at% (purity 99.9%) targets (Kurt J. Lesker Company, Pittsburgh, PA, USA). Prior to the deposition, the substrates: conducting glass—indium tin oxide ITO (Sigma-Aldrich, Inc., St. Louis, MO, USA), fused silica JGS-1 (Continental Trade, Warszawa, Poland) and crystalline (100)-oriented silicon (Si-Mat Silicon Materials, Kaufering, Germany) were sonicated for 10 min in each solution: water with an addition of detergent, deionized water, acetone and isopropanol. After drying, the substrates were mounted in the load-lock chamber (pressure below 2 × 10^−6^ mbar) and cleaned with the ion-gun operating in an argon atmosphere with a beam energy 3 kV and emission current 10 mA. Deposition of thin films was carried out in the process chamber under controlled technological conditions, the details of which are given in Table 1. Distance between the target surface and substrates was set as 75 mm. The sample holder was heated up to 350 °C at a rate of 10 °C/min and kept at this temperature during the process. Continuous rotation of the sample holder (1 rpm) was provided. To ensure proper sputtering pressure and modify the composition of thin films, the total flow rate was kept at 40 sccm, and argon to oxygen flow rates were adjusted. The O_2_ content in atmosphere equal to 15% corresponds to argon to oxygen flow rate Ar:O_2_ = 34 sccm:6 sccm, and 20% of oxygen means Ar:O_2_ = 32 sccm:8 sccm. After two-stage presputtering (10 min in Ar at 100 W and 20 min in Ar + O_2_ at 200 W), deposition was carried out for 50 min with the sputtering power of 200 W.

Morphology, structural, optical and electronic properties were determined from the Scanning Electron Microscopy SEM (Helios G4 PFIB CXe DualBeam, Thermo Fischer Scientific, Waltham, MA, USA, equipped with Quanta XFlash 630 EDS detector, Bruker, Billerica, MA, USA), X-ray diffraction XRD (X’Pert MPD diffractometer, Philips, now Malvern Panalytical, Malvern, UK), optical spectrophotometry (Lambda 19 spectrophotometer, Perkin-Elmer, Inc., Waltham, MA, USA) and X-ray Photoelectron Spectroscopy XPS (PHI5000 VersaProbe II Scanning XPS Microprobe, Physical Electronics PHI, Chigasaki, Japan). The XPS analyses were carried out in the system using monochromatic Al Kα (1486.6 eV) X-rays focused to a 100 µm spot and scanned over the area of 400 µm × 400 µm. All XPS spectra were charge referenced to the unfunctionalized, saturated carbon (C–C) C 1s peak at 285.0 eV. Deconvolution of spectra was carried out using MultiPak software (v.9.9.0.8, 2017, PHI, Chigasaki, Japan). XRD results were analyzed according to anatase 01-078-2486, rutile 01-086-0147 and Er_2_Ti_2_O_7_ 01-073-1700 cards. Luminescence properties were measured by WITec spectrometer equipped with a 488 nm laser diode (50 mW, 6.7 mW/µm^2^) and CCD UV-NIR detector (WITec Wissenschaftliche Instrumente und Technologie GmbH, Ulm, Germany) as well as Horiba QuantaMaster-80450-22 fluorometer (Horiba Canada Inc, Ontario, Canada) equipped with 350 mm double monochromator, TE-cooled photon counting PMT R928 (Hamamatsu Photonics, Hamamatsu, Japan) combined with liquid N_2_ cooled InGaAs solid detectors under 980 nm (1 W) diode-pumped solid-state DPSS laser excitation (CNI, Changchun, China).

## 3. Results and Discussion

**Figure 1:**
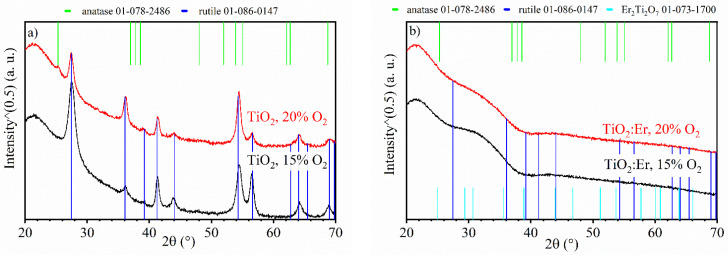
XRD patterns recorded for the thin films of: (**a**) pure TiO_2_ deposited at 15% O_2_ (black curve) and 20% O_2_ (red curve) and (**b**) Er-doped TiO_2_ deposited at 15% O_2_ (black curve) and 20% O_2_ (red curve). Positions of reference peaks are marked by vertical lines. Reference patterns are from anatase 01-078-2486, rutile 01-086-0147 and Er_2_Ti_2_O_7_ 01-073-1700 cards.

The XRD patterns recorded for thin films are presented in Figure 1. Undoped titanium dioxide thin films under these slightly nonstoichiometric conditions (15% and 20% O_2_) crystalized mostly as rutile (Figure 1a). However, at higher content of oxygen in the Ar + O_2_ mixture (20% O_2_), the onset of anatase precipitation was detected. At 2θ = 25.3° XRD line, which corresponds to the (101) plane of anatase (the reflex with the highest intensity) can be seen. For rutile, some differences in levels of signal intensities between TiO_2_ 15 and 20% O_2_, and relative intensities from the 01-078-2486 card, were observed (Figure 1a). Thin films grow with crystallites not randomly oriented like grains in powder samples. Preferred orientation usually occurs. In the XRD setup, some crystallographic planes might be preferentially exposed to the incoming beam, and thus the intensity of the signal is higher.

In contrast to undoped TiO_2_, X-ray diffraction patterns of TiO_2_:Er thin films are featureless, indicating probable amorphization (Figure 1b). This effect was reported [33] and attributed to a significantly larger ionic radius of Er^3+^ ions (0.0890–0.1214 nm) than that of Ti^4+^ (0.042–0.074 nm). At a high concentration of Er^3+^, formation of erbium titanate pyrochlore structure, Er_2_Ti_2_O_7_ was observed [21] for samples annealed at a high temperature exceeding 800 °C. Significant increase in the X-ray intensity for 2θ between 28° and 38° occurs as seen in Figure 1b. To investigate the nature of this effect, the XRD patterns of amorphous silica substrate were collected. Then, the substrate pattern was multiplied by an empirical factor and subtracted from the pattern recorded for films. As a result, a broad peak with a maximum located at 21.5° (related to the amorphous substrate) was eliminated. This operation has put in evidence a wide feature, the maximum of which is located at around 30.5°. It is worth noticing that the reflex of the highest intensity from (222) planes of cubic Er_2_Ti_2_O_7_ occurs at 2θ = 30.7°. It may suggest the beginning of crystallization of nanosized Er_2_Ti_2_O_7_ or at least the presence of short-range order. Exemplary results before and after correction are presented in the Supplementary Materials, Figure S1.

**Figure 2:**
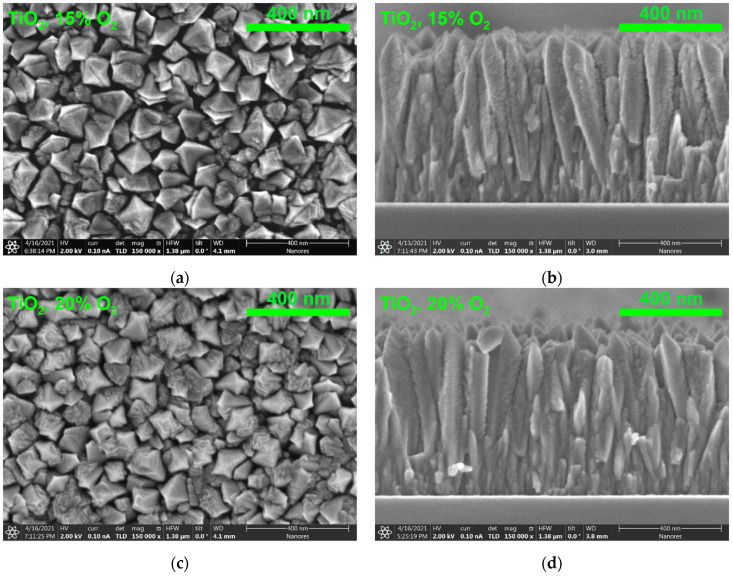
SEM images of the surface and cross-section of undoped TiO_2_ thin films deposited at (**a**,**b**) 15% O_2_ and (**c**,**d**) 20% O_2_.

**Figure 3:**
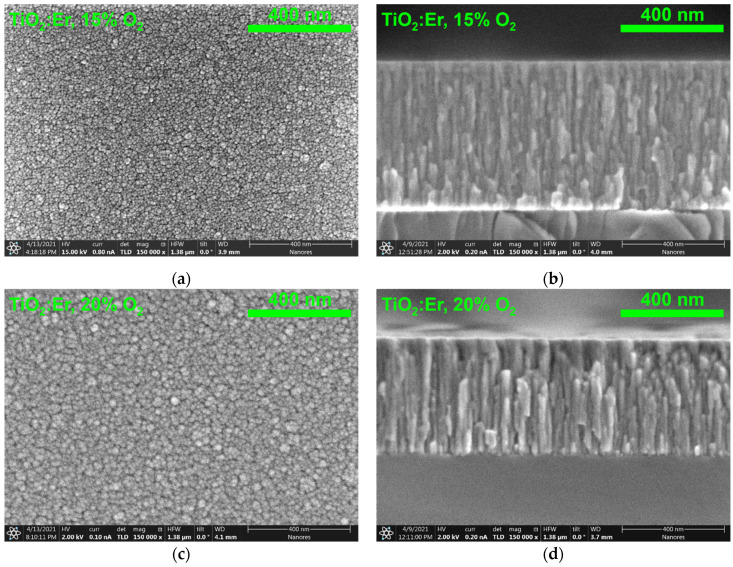
SEM images of the surface and cross-section of TiO_2_:Er thin films deposited at (**a**,**b**) 15% O_2_ and (**c**,**d**) 20% O_2_.

The SEM images (Figure 2 and Figure 3) show that the substrates were homogeneously covered with thin films. An analysis of the surface and cross-sectional morphology of thin films confirms the conclusions drawn from the XRD studies. Well-crystalized pure TiO_2_ layers grow as well-shaped tetragonal columns with approximately 660 nm height (Figure 2b,d). The TiO_2_ layer’s surface is rough due to the pyramid-like tops of columns (Figure 2a,c). TiO_2_ columns have a tendency to enlarge their diameter with height and then to narrow due to the formation of the pyramid-like top.

In the case of TiO_2_ thin films containing Er, a smooth surface and compact growth of narrow fibers were obtained (Figure 3). A small change in the surface morphology is observed with the oxygen concentration during deposition; a higher amount of oxygen (20% O_2_; Figure 3c) leads to larger grains at the surface in comparison to grains at the surface of 15% O_2_ layer (Figure 3a). Moreover, thin fibers shown in Figure 3b for 15% O_2_ changed to thicker columns (Figure 3d) for 20% O_2_.

The grain analysis based on SEM images presented in Figure 2 and Figure 3 was carried out. The details concerning the distribution of the grain size can be found in the Supplementary Materials (Table S1). The average grain size of undoped TiO_2_ thin films is about 90–95 nm and does not depend on the oxygen concentration during sputtering. The average grain size of TiO_2_:Er films deposited at 15% O_2_ is about 14 nm and increases slightly to 19 nm for 20% O_2_.

**Figure 4:**
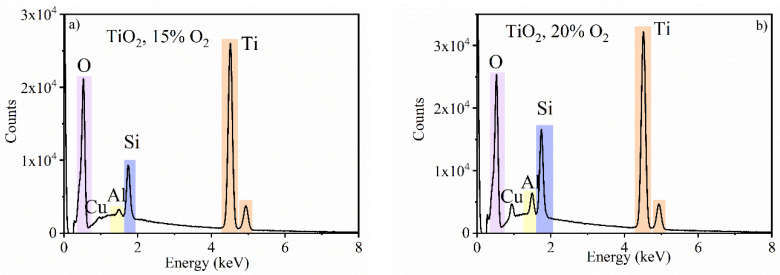

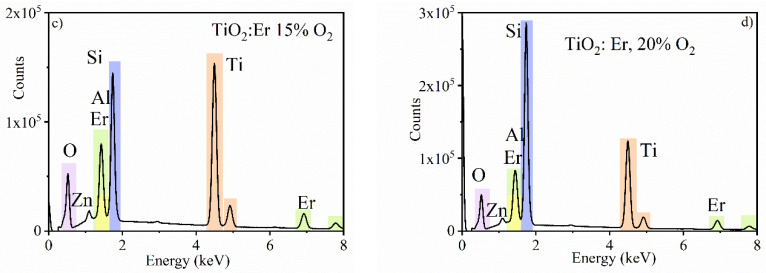
EDS analysis of TiO_2_ thin films: undoped TiO_2_ deposited at: (**a**) 15% O_2_ and (**b**) 20% O_2_; and TiO_2_:Er deposited at: (**c**) 15% O_2_ and (**d**) 20% O_2_.

The elemental composition of thin films was studied by the Energy Dispersive Spectroscopy EDS. The EDS spectra for pure TiO_2_ and TiO_2_:Er are presented in Figure 4. The measurement confirmed that all layers are composed of titanium and oxygen (Figure 4a–d). Moreover, for TiO_2_:Er samples (Figure 4c,d), peaks from Er (1.4, 6.9 and 7.8 keV) appeared. In all EDS spectra, a high-intensity peak assigned to Si originated from the substrate because the beam penetration depth was bigger than that of the thin film thickness. Additionally, due to the small size of samples, the signals from elements in an aluminum sample holder, copper clips and brass screws were collected. Thus, weak signals at 0.9, 1.0 and 1.5 keV can be attributed to copper, zinc and aluminum, respectively.

**Figure 5:**
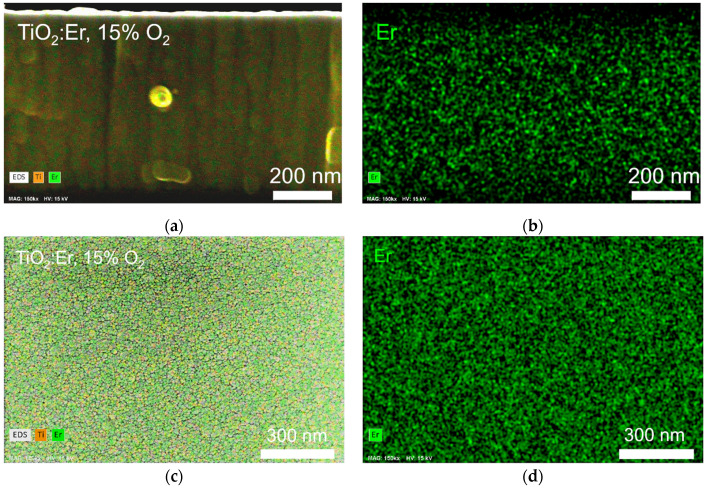
EDS elemental mapping: (**a**,**b**) cross section and (**c**,**d**) surface of TiO_2_:Er deposited at 15% O_2_.

EDS elemental mapping for TiO_2_:Er deposited at 15% of O_2_ (Figure 5) demonstrates the homogenous distribution of erbium in the bulk (cross-section image; Figure 5a,b) and on the surface (Figure 5c,d) of thin films.

**Figure 6:**
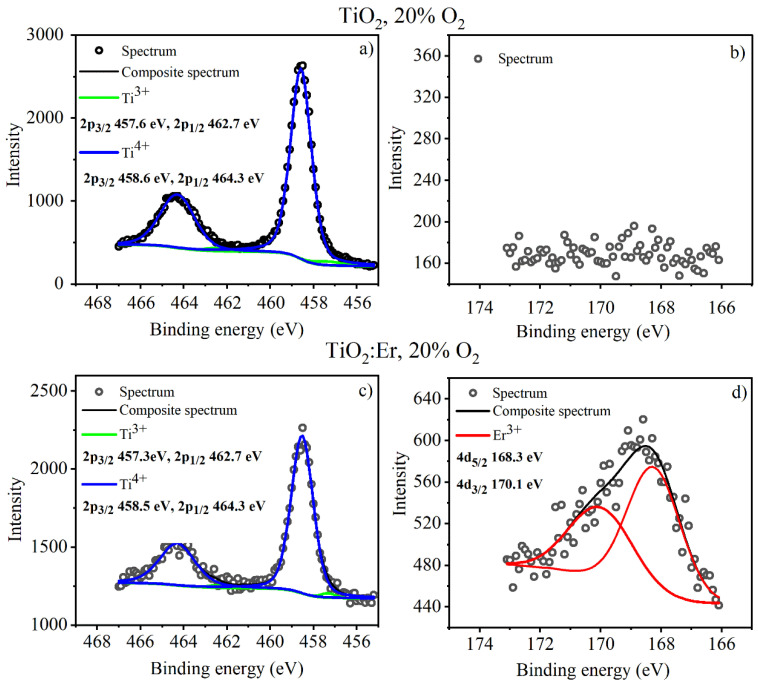
XPS spectra for the thin films of: (**a**,**b**) TiO_2_—deposited at 20% O_2_, and (**c**,**d**) TiO_2_:Er—deposited at 20% O_2_.

The XPS spectra of pure and Er-doped TiO_2_, deposited at 20% O_2,_ are shown in Figure 6 within a region of binding energies covering Ti 2p (Figure 6a,c) and Er 4d peaks (Figure 6b,d). The Ti 2p spectrum was fitted with two 2p doublet structures (p_3/2_–p_1/2_ doublet separation equals 5.2 eV for Ti^3+^ state and 5.7 eV for the Ti^4+^ state) originating from two different states of Ti, which indicates the presence of nonstoichiometric titanium dioxide. Titanium is found mainly in Ti^4+,^ which appears as a 2p_3/2_ line centered at 458.6 eV, but there is also a low concentration of Ti^3+^ oxidation state, which is expressed with 2p_3/2_ line at 457.3 eV [34].

The Er 4d spectrum was fitted with one doublet structure (d_5/2_–d_3/2_ doublet separation equals 2.05 eV) with main 4d_5/2_ centered at 168.3 eV, which indicates Er^3+^ oxidation state [35]. Table S2 in the Supplementary Materials contains the results of the XPS data fitting (peak position, its full width at half maximum FWHM and the area).

The composition of thin films was determined from XPS data presented in Table S2. The ratio of Er/(Er + Ti) derived from the analysis of the XPS peak area is about 0.14. As we demonstrated the uniform distribution of Er and Ti in the films (cross-sectional images presented in Figure 5a,b), it is reasonable to assume that this value represents not only the surface composition but that of the bulk as well.

**Figure 7:**
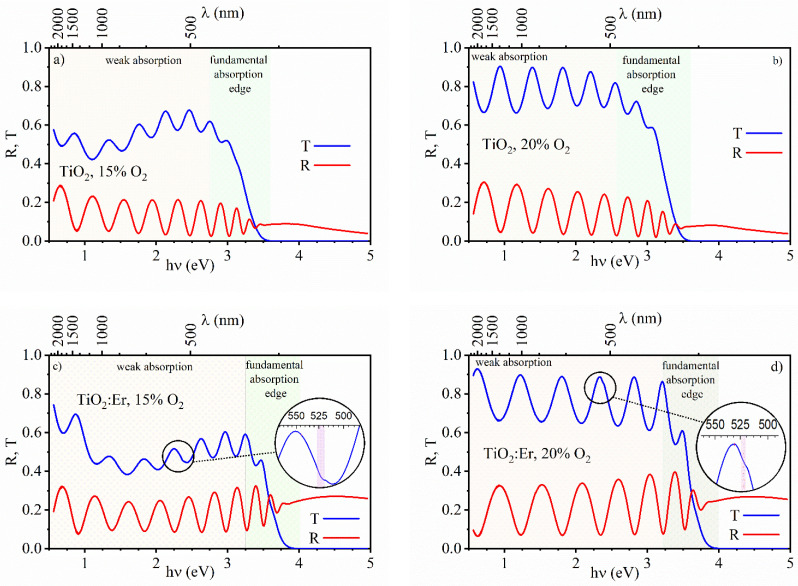
Spectral dependence of the transmittance T and reflectance R coefficients of thin films: undoped TiO_2_ (**a**) 15% O_2_ and (**b**) 20% O_2_; TiO_2_:Er (**c**) 15% O_2_ and (**d**) 20% O_2_. Additional absorption features zoomed-in circles.

The results of spectrophotometric measurements of the transmittance T and reflectance coefficient are presented in Figure 7. Regions of a weak absorption and fundamental absorption edge are marked. In the weak absorption region, the characteristic oscillations of T and R can be seen. They are related to the light interference at two interfaces: air–thin film and thin film–substrate. This behavior is very well known and described in many fundamental handbooks [36,37,38].

For a light incident on a film deposited onto a non-absorbing substrate, from the spectral dependence of the transmittance T and reflectance R coefficients over a weak absorption region, one can derive absorption coefficient and refractive index using a method described in detail in [39].

The transmittance coefficient of thin films deposited at 20% O_2_ is high and reaches 90% level for both TiO_2_ and TiO_2_:Er. Lower content of oxygen (15%) results in a decrease in T to approximately 65–60%. Furthermore, over the near-infrared range (at about 1000 nm), an additional extended minimum in T develops when thin films are deposited at 15% O_2_. The increased absorption within this spectral range could be explained by the departure from stoichiometry and increased concentration of defects such as oxygen vacancies [40,41].

The erbium-modified films (Figure 7c,d) exhibit additional absorption features marked with a circle and zoomed in. This additional absorption occurs at the wavelength of 525 nm, which corresponds to the strongest erbium absorption band. It might be evidence of erbium incorporation into TiO_2_ crystal lattice. Here, this effect is barely visible due to a relatively low amount of erbium in thin films with low thickness in comparison to powder samples (bulk material) with a similar concentration of erbium. This suggests that in the case of thin films, photoluminescence measurements are necessary to study the influence of lanthanides incorporated into TiO_2_. Within the region of the fundamental absorption edge, the transmittance coefficient abruptly drops to zero with the increasing photon energy hν. This effect is due to the band gap E_g_ of TiO_2,_ which falls within the UV range of the light spectrum.

**Figure 8:**
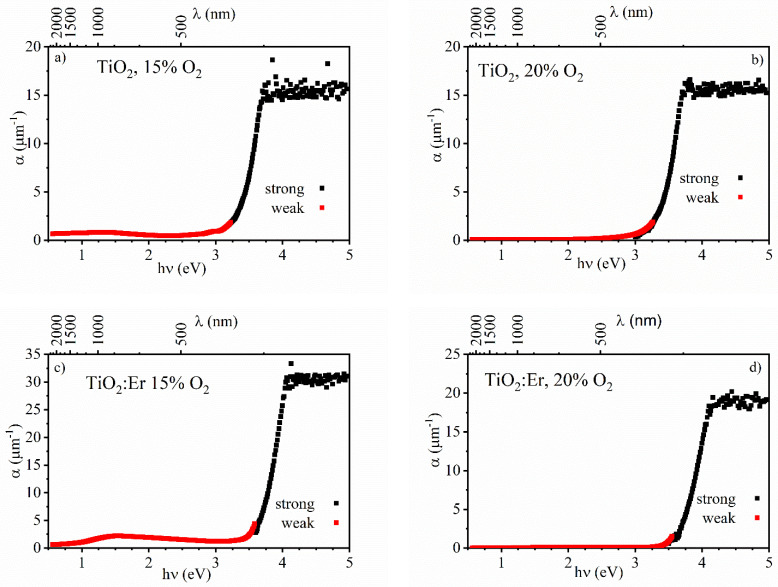
Spectral dependence of the absorption coefficient α of thin films: undoped TiO_2_ (**a**) 15% O_2_ and (**b**) 20% O_2_, and TiO_2_:Er (**c**) 15% O_2_ and (**d**) 20% O_2_.

The absorption coefficient α was calculated independently in the weak and strong absorptions regions. Strong absorption appears close to the fundamental absorption edge. In the first step, the “envelope method” for transmittance and reflectance spectra was applied (exemplary results can be found in the Supplementary Materials, Figure S2. Then, the spectral dependence of the absorption coefficient within the weak and strong absorption region was determined (Figure 8). In Figure 8a,c, an increased light absorption over the VIS and NIR range for 15% O_2_ in comparison to 20% is shown.

**Figure 9:**
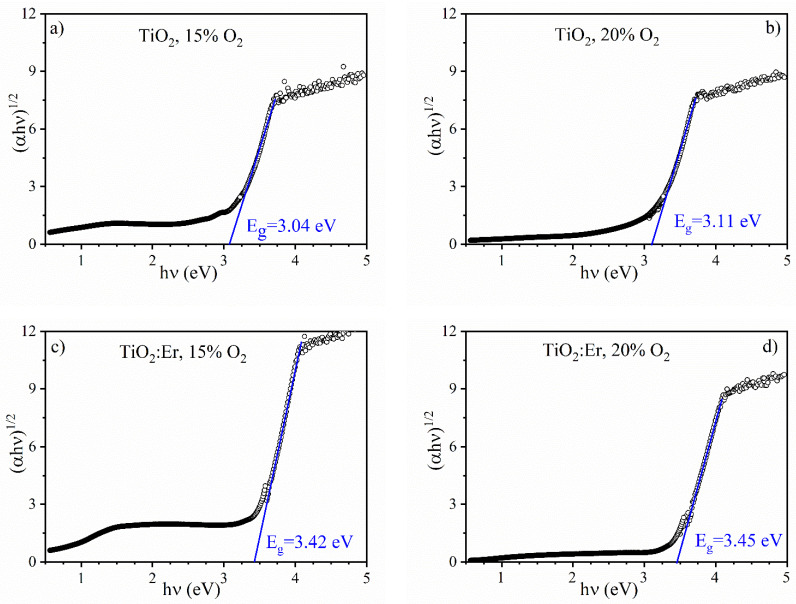
Band gap E_g_ determination for thin films—spectral dependence of the (αhν)^1/2^ coefficient vs. photon energy hν: undoped TiO_2_ (**a**) 15% O_2_ and (**b**) 20% O_2_; TiO_2_:Er (**c**) 15% O_2_ and (**d**) 20% O_2_.

The band gap energy E_g_ was determined from the cut-off of plots of (αhν)^1/2^ versus the photon energy hν at the zero-level, as shown in Figure 9. The band gap energy of undoped TiO_2_ is E_g_ = 3.04 eV for 15% O_2_ and 3.11 eV for 20%. The values for lower oxygen content correspond to the rutile band gap (3.0 eV), and for higher oxygen content, it is an effect of beginning the crystal phase transition from rutile to anatase (3.2 eV).

Such a shift of the band gap energy obtained from the analysis of the optical spectra is expected because the E_g_ of about 3.0 eV for rutile and of about 3.2 eV for anatase is usually reported [42]. Actually, the experimental uncertainty being of the order 0.01–0.02 eV is much smaller than the reported shift of 0.07 eV and can be explained by changes in the film crystallization, as suggested in Figure 1a.

However, in the case of TiO_2_:Er, the band gap energy E_g_ is certainly much bigger (3.42–3.45 eV) and does not depend so much on the oxygen content during the deposition of thin films. This significant blue shift in the fundamental absorption edge can be ascribed to the size-effect as a much smaller grain size was obtained for TiO_2_:Er. Nevertheless, the possibility of precipitation of nanosized crystals of the secondary phase Er_2_Ti_2_O_7_ cannot be excluded. The size of the nanocrystals being smaller than 5 nm remains in agreement with the extended uplift in the XRD intensity around 2θ = 30° (a broad feature appearing in Figure 1b and Figure S1).

There is not enough experimental evidence concerning the band gap of Er_2_Ti_2_O_7_. According to [43], the band gap of Er_2_Ti_2_O_7_ is about 3.39 eV, which coincides reasonably well with our observation. However, due to the lack of direct proof, the onset of Er_2_Ti_2_O_7_ precipitation remains a hypothesis in our work.

**Figure 10:**
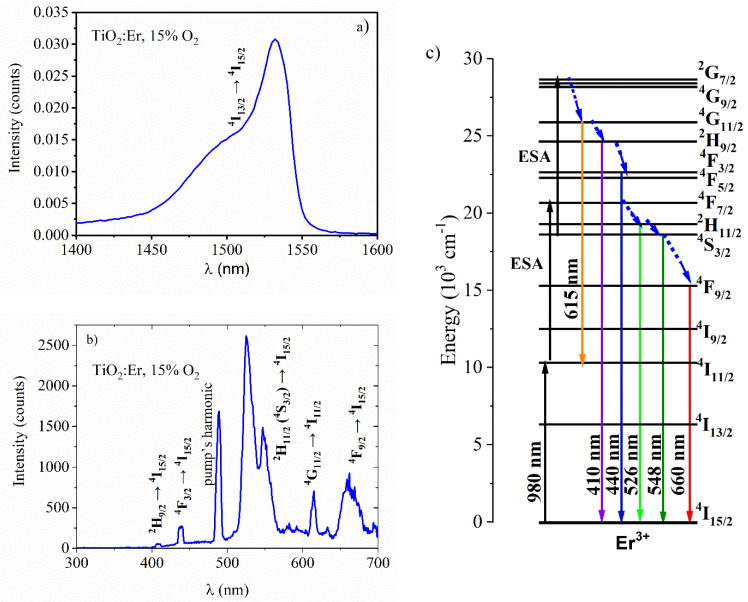
Luminescent properties of Er-doped TiO_2_ thin films deposited at 15% O_2_ excited with 980 nm laser: (**a**) photoluminescence spectrum, (**b**) up-conversion spectrum, (**c**) energy diagram in Er^3+^.

The luminescence properties of the active layers were measured in the UV-NIR range under the laser diodes at 980 nm and 488 nm wavelength excitation. In the first case (Figure 10a), a strong emission located around 1550 nm can be seen, which results from the typical ^4^I_13/2_ → ^4^I_15/2_ transition in erbium ions. The luminescence presented in Figure 10b reveals the up-conversion mechanism upon excitation at 980 nm. The emission peaks observed in the spectrum can be attributed to the following transitions in Er^3+^: 410 nm (^2^H_9/2_ → ^4^I_15/2_), 440 nm (^4^F_3/2_ → ^4^I_15/2_), 526–548 nm (^2^H_11/2_ (^4^S_3/2_) → ^4^I_15/2_) and 660 nm (^4^F_9/2_ → ^4^I_15/2_), as indicated in the scheme in Figure 10c. These emission peaks are possibly due to the three-photon excitation process described by ^4^I_15/2_ → ^4^I_11/2_ → ^4^F_7/2_ → ^2^G_7/2_. The excitation of the ^2^G_7/2_ level follows the non-radiative relaxation from the ^4^F_7/2_ level to the thermally coupled ^2^H_11/2_ and ^4^S_3/2_ levels. The absorption schemes were discussed in different low and high phonon environments as they determine luminescent transitions in RE ions [44]. The important aspect also concerns the cross-relaxation (CR) mechanisms in highly doped Er materials, which also can lead to the UP emission such excited state absorption (ESA) [45]. It might also promote other luminescent transitions, such as that at 615 nm (^4^G_11/2_ → ^4^I_11/2_) presented in Figure 10b, which was only registered in the fluoride low phonon materials (ZrF_4_-BaF_2_-LaF_3_-AlF_3_-NaF ZBLAN glass, NaYF_4_ crystals) [44]. The origin of the 490 nm band is related to the second harmonic of the 980 nm pump.

**Figure 11:**
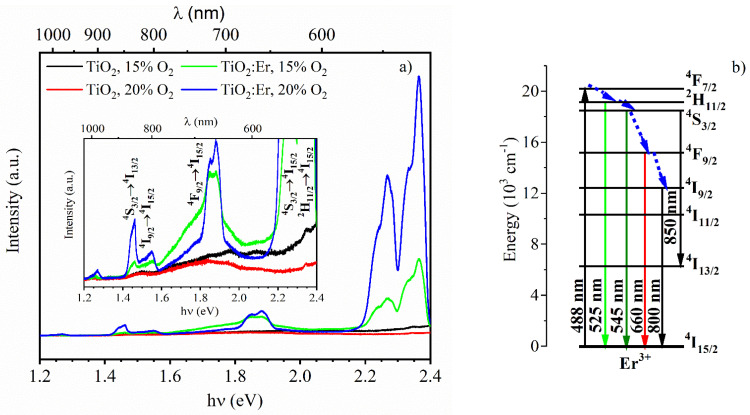
(**a**) Luminescence spectrum of pure TiO_2_ and Er-doped TiO_2_ thin films deposited at 15 and 20% of O_2_ (enlargement in the inset) excited with 488 nm laser; (**b**) simplified diagram of possible transitions in Er^3+^.

However, it is worth noting that the high concentration of Er ions induces the complex CR processes where energy transfer within the TiO_2_ band gap may not be excluded. When analyzing this aspect, the samples were excited at 488 nm, which led to the direct excitation of ^4^F_7/2_ erbium energy level and also corresponded to the sub-band excitation in TiO_2_. Under these conditions, the luminescence spectrum was registered within the wavelength range of 500–1100 nm, including undoped TiO_2_ samples for comparison (Figure 11a). When analyzing the erbium emission spectrum in both cases (15% and 20% O_2_), the characteristic intense transitions of ^2^H_11/2_ (^4^S_3/2_) → ^4^I_15/2_ and ^4^F_9/2_ → ^4^I_15/2_, assigned to the emission in the green (525 nm, 550 nm) and red (660 nm) part of the spectrum, were observed (Figure 11b). Moreover, in the NIR range, the luminescent bands at the wavelength of 800 nm (^4^I_9/2_ → ^4^I_15/2_), 850 nm (^4^S_3/2_ → ^4^I_13/2_) and much weaker at 980 nm (^4^I_11/2_ → ^4^I_15/2_) were recorded. By analyzing the spectra of undoped TiO_2_ thin films, the presence of bands in the range of 500–550 nm (2.48–2.25 eV, max. intensity 530 nm–2.5 eV), 580–725 nm (2.14 eV–1.7 eV), 840 nm (1.48 eV), 990 nm (1.25 eV) and 1050 nm (1.18 eV) was revealed. Green and red emissions are usually associated with the presence of oxygen vacancies and Ti^3+^ defects, respectively [46,47]. Oxygen vacancies can be located in a wide range of states (mid-gap), i.e., 2.269 eV (447 nm) to 2.719 eV (456 nm). However, in this case, radiation transitions from the TiO_2_ conduction band would have to take place, which is relatively unlikely, taking into account the 488 nm excitation. On the other hand, the visible luminescence (500–530 nm) may originate from the Ti^3+^ energy transfer to the surface oxygen vacancies [46,48]. The next two bands, i.e., at 840 nm and 990 nm, are related in particular to the emission in rutile TiO_2_ and the occurrence of self-interstitial defects of Ti^4+^ (Ti^3+^) [49].

Finally, the aspect of energy transfer between Er ions and the mid-gap levels of TiO_2_ should be mentioned here. Figure 11a shows a strong, wide red band from TiO_2_ overlapping with the emission of the 660 nm transition within the erbium energy bands. It may suggest the possible energy transfer from Er bands to TiO_2_ trap bands. Although it is not unequivocal at this stage, it proves the lack of clustering of erbium ions and the suitability of these layers in the up-conversion systems.

## 4. Conclusions and Perspectives

Optically active matrices such as fluorides and silicates doped with lanthanide ions are very well-known for efficient visible-to-UV [50] or NIR-to-UV [51] up-conversion. However, it was recognized that the incorporation of rare earth RE^3+^ ions into the TiO_2_ host becomes a big challenge because of the incompatibility of the ionic radii of RE^3+^ and Ti^4+^. Dordević et al., in review [23] on RE elements in anatase TiO_2_ nanoparticles, even used the term “unusual” with respect to the TiO_2_ matrix. Nevertheless, due to its excellent stability, TiO_2_ remains one of the most promising photocatalysts and candidates for photoanodes in water splitting. Many efforts focused on Er^3+^ ion incorporation in TiO_2,_ as shown in an excellent review of Mazierski et al. [52]. Three other lanthanide ions, Ho^3+^, Tm^3+^ and Nd^3+^, incorporated in TiO_2_ anatase and their role in photocatalysis were discussed. It was pointed out that theoretical investigations by the density functional theory DFT method are quite scarce for these systems. Moreover, according to [52], there is no reliable proof that the up-conversion process has a real effect on improvement in photocatalytic activity.

In our work, comprehensive studies on TiO_2_ thin films with embedded Er^3+^ were undertaken. This system is known to demonstrate up-conversion from NIR (980 nm) to the visible range of the light spectrum (525–545 nm), and it is expected that by a three-photon nonlinear absorption, the UV range (390–410 nm) can be reached [53].

In conclusion, it was demonstrated that thin films of TiO_2_:Er deposited by magnetron sputtering:

- Contain a high amount of Er (14 at.%);
- Are amorphous to a large extent, but precipitation of the secondary phase of Er_2_TiO_7_ cannot be excluded;
- Are smooth with the grain size much smaller than that of undoped TiO_2_;
- Have the band gap energy much larger (0.4 eV blue shift) than that of undoped TiO_2_;
- Exhibit up-conversion upon illumination at 980 nm;
- Exhibit photoluminescence in the VIS and NIR range of the light spectrum upon excitation at 488 nm.

In the near future, it is intended to perform photoelectrochemical measurements with planar thin films photoanodes of TiO_2_:Er in order to assess whether there is a correlation between the efficiency of hydrogen generation and photoluminescent properties. The optimization of Er concentration will be accompanied by the incorporation of Yb playing the role of sensitizer.

## Figures and Tables

**Table 1:** Parameters of thin films deposition process.

Sample	Target	O_2_ Content in Ar + O_2_	Thickness ^1^ (nm)	Deposition Rate (nm/min)
TiO_2_, 15% O_2_	Ti	15%	640	12.8
TiO_2_, 20% O_2_	Ti	20%	610	12.2
TiO_2_:Er, 15% O_2_	Ti/Er (90/10 at%)	15%	550	11.0
TiO_2_:Er, 20% O_2_	Ti/Er (90/10 at%)	20%	480	9.6
Total flow rate 40 sccm; substrate temperature 350 °C; base pressure 10^−7^–10^−8^ mbar; sputtering pressure 6.3 × 10^−3^ mbar; sputtering power 200 W				

^1^ Thickness measured by a stylus profilometer (Talystep, Taylor Hobson, Leicester, UK).

## Data Availability

The data presented in this study are available on request from the corresponding author. The data are not publicly available due to the author’s readiness to provide it on request.

## Supplementary Materials

The following are available online at https://www.mdpi.com/article/10.3390/ma14154085/s1, Figure S1: XRD patterns (as measured and after correction) recorded for the Er-doped thin films deposited at 15% O_2_ (black curve) and 20% O_2_ (red curve). A correction was performed by subtraction of the recorded substrate pattern. Positions of reference peaks are marked with vertical lines. Reference patterns are from anatase 01-078-2486, rutile 01-086-0147, and Er_2_Ti_2_O_7_ 01-073-1700 cards; Table S1: Grain size analysis; Table S2: XPS results analysis; Figure S2: Optical properties analysis of TiO_2_ thin films deposited at 20% O_2_—spectral dependence of: (a) the transmittance T and reflectance R coefficients with envelopes; (b) the calculated absorption coefficient α.
