# Supplementary material for: Optically Active TiO2:Er Thin Films Deposited by Magnetron Sputtering
Source: Materials (Basel). 2021 Jul 22;14(15):4085. doi: 10.3390/ma14154085 (PMC8348420; doi:10.3390/ma14154085)
Supplement: Supplementary file 1 [file materials-14-04085-s001.zip › materials-1281091-supplementary.pdf]

materials
MDPI

Supplementary Materials

Optically Active TiO_2:Er Thin Films Deposited by Magnetron Sputtering

Anna Kot [1,*], Marta Radecka [1], Dominik Dorosz [1] and Katarzyna Zakrzewska [2]

[1] Faculty of Materials Science and Ceramics, AGH University of Science and Technology, al. A. Mickiewicza 30, 30-059 Kraków, Poland; radecka@agh.edu.pl (M.R.); ddorosz@agh.edu.pl (D.D.)
[2] Faculty of Computer Science, Electronics and Telecommunications, AGH University of Science and Technology, al. A. Mickiewicza 30, 30-059 Kraków, Poland; zak@agh.edu.pl
* Correspondence: annakot@agh.edu.pl

Citation: Kot, A.; Radecka, M.; Dorosz, D.; Zakrzewska, K. Optically Active TiO2:Er Thin Films Deposited by Magnetron Sputtering. *Materials* **2021**, *14*, 4085. https://doi.org/10.3390/ma14154085

Academic Editor: Roberta G. Toro

Received: 15 June 2021
Accepted: 14 July 2021
Published: 22 July 2021

Publisher's Note: MDPI stays neutral with regard to jurisdictional claims in published maps and institutional affiliations.

Copyright: © 2021 by the authors. Licensee MDPI, Basel, Switzerland. This article is an open access article distributed under the terms and conditions of the Creative Commons Attribution (CC BY) license (http://creativecommons.org/licenses/by/4.0/).

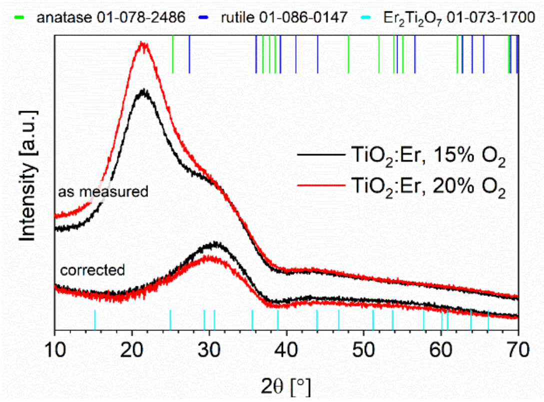

Figure S1. XRD patterns (as measured and after correction) recorded for the Er-doped thin films deposited at 15% O_2 (black curve) and 20% O_2 (red curve). Correction was done by subtraction of recorded substrate pattern. Positions of reference peaks are marked with vertical lines. Reference patterns are from anatase 01-078-2486, rutile 01-086-0147, and $Er_2Ti_2O_7$ 01-073-1700 cards.

Table S1. Grain size analysis.

	15% O_2	20% O_2
	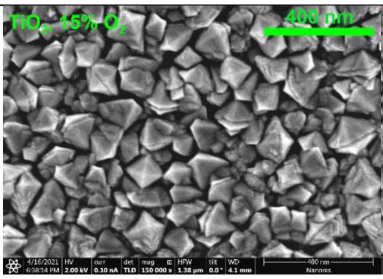	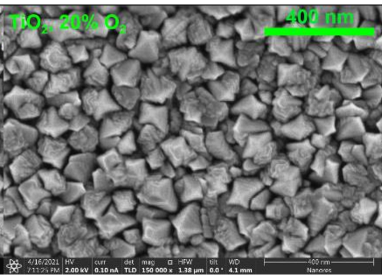
TiO_2	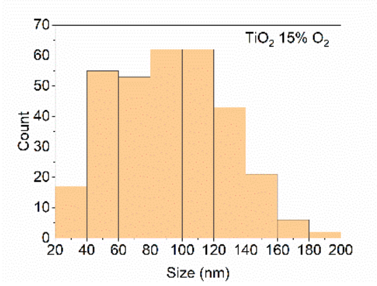	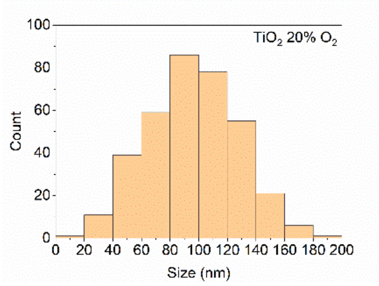
N total	321	357
Mean	92.44 nm	96.13 nm
Standard deviation	34.97 nm	31.81 nm
**Average grain size**	**92±35 nm**	**96±32nm**
	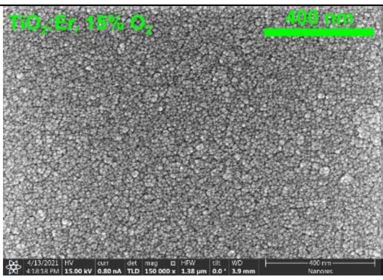	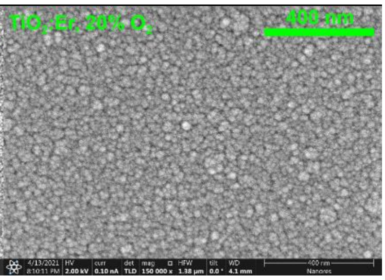
TiO_2:Er	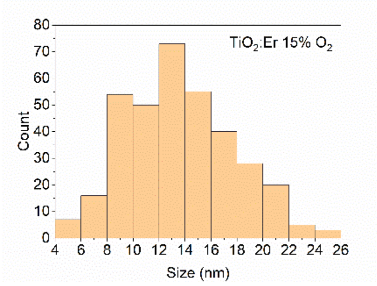	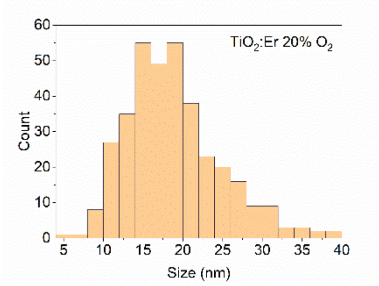
N total	351	356
Mean	13.61 nm	18.84 nm
Standard deviation	4.11 nm	5.92 nm
**Average grain size**	**14±4 nm**	**19±6 nm**

Table S2. XPS results analysis.

	Peak position (eV)	Area	FWHM
	TiO_2, 20% O_2		
Ti^{3+}: $2p_{3/2}$	457.6	237.0	2.73
Ti^{3+}: $2p_{1/2}$	462.7	71.6	1.45
Ti^{4+}: $2p_{3/2}$	458.6	2594.3	0.96
Ti^{4+}: $2p_{1/2}$	464.3	2060.4	2.34
	TiO_2:Er, 20% O_2		
Ti^{3+}: $2p_{3/2}$	457.3	38.6	1.13
Ti^{3+}: $2p_{1/2}$	462.7	18.0	0.96
Ti^{4+}: $2p_{3/2}$	458.5	1211.0	1.00
Ti^{4+}: $2p_{1/2}$	464.3	856.5	2.38
Er^{3+}: $4d_{5/2}$	168.3	190.8	1.46
Er^{3+}: $4d_{3/2}$	170.1	343.3	3.04

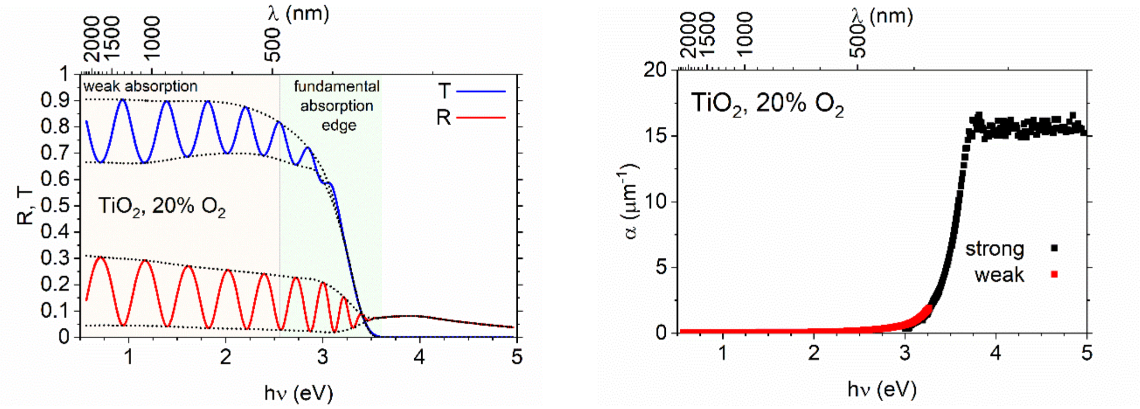

(**a**) (**b**)

Figure S2. Optical properties analysis of TiO_2 thin films deposited at 20% O_2 - spectral dependence of: (a) the transmittance T and reflectance R coefficients with envelopes; (b) the calculated absorption coefficient α.
